# Case in Which the Substance of the Uterus Was in a Great Measure Destroyed during Pregnancy; with an Account of the Appearances on Dissection

**Published:** 1787

**Authors:** William Blackburne

**Affiliations:** Member of the Royal College of Physicians, London


VI. *Caſe in which the Subſtance of the Uterus was in a great Meaſure deſtroyed during Pregnancy; with an Account of the Appearances on Diſſection. Communicated in a Letter to Dr.* Simmons *by* William Blackburne, *M. D., Member of the Royal College of Phyſicians, London.*

ABOUT the beginning of April, 1784, I was deſired to viſit a poor woman who ſuffered under the following ſymptoms: — She complained of violent pain in the abdomen, back, and thighs; of conſtant thirſt, ſhiverings, followed by heat and profuſe ſweats; her nights were reſtleſs, her breathing difficult; and ſhe was alſo affected with dyſuria and coſtiveneſs. Her pulſe was hard and quick; ſhe had no appetite; and preſented the moſt horrid ſpectacle of emaciation I ever beheld. A hard unequal tumour occupied the whole abdomen, which, on being gently agitated, produced an evident evident fluctuation; and there was a conftant, fœtid, ichorous difcharge from the vagina.

In fo hopelefs a cafe palliatives alone were indicated. Thefe were fo adminiftered as, in fome degree, to alleviate her mifery.

In the courfe of two or three vifits I obtained from my unhappy patient the fubfequent relation:—According to her reckoning, fhe had arrived at the eleventh month of her utero-geftation, and had happily paffed through the regular gradations of pregnancy until the conclufion of the feventh month, when, immediately after raifing a pail of water to her head, fhe was attacked with a moft violent pain in the abdomen, which continued for the fpace of thirty-fix hours, and was at length removed by bleeding at the arm and other antiphlogiftic remedies. During feveral weeks after this accident fhe felt great pain in the lower part of her belly whenever the child, which had quickened about the fourth month, moved, and on thefe occafions the child feemed to her to rife up as high as the epigaftric region. In this ftate the poor woman continued until about a week before the natural period of labour, when her breafts became full of milk, and a fmall quantity of blood and mucus flowed from the vagina. The milk deferted the breafts, and the uterine difcharge ceafed, both at the fame time, after a very fhort continuance. At the completion of the nine months, regular, but very gentle, labour pains came on; thefe being inefficient, were appeafed by a proper dofe of laudanum, the attending furgeon judging them to be only what are ftiled grinding pains. Nearly in the courfe of a week the fame kind of pain recurred; but was now conftant, inalternate, and particularly affected the back and iliac regions. At this time alfo a flight colouring iffued from the vagina, and a copious flow of milk from the mammæ. This fort of pain was removed too by laudanum, and never returned.

Under circumftances fo ftriking and uncommon, I could not truft to my own judgement, efpecially as I did not practife midwifery, and therefore requefted the opinions of the moft eminent of that profeffion in Durham. On examination, the os uteri was found almoft clofe to the orifice of the vagina. It was the general fentiment that there did exift a fœtus, but whether extra-uterine or not could not be determined. It was agreed that the patient was too much reduced to undergo any operation; tion; and that ſhe muſt be left to nature in a great meaſure, only perſevering in the mild and palliative means which had been previouſly recommended.—I continued my attendance.

On the 8th of May my patient diſcharged by ſtool four quarts of very offenſive purulent matter, mixed with blood. Moſt evident relief of all the hectic ſymptoms ſucceeded to this evacuation; her appetite was much improved, and the tumour of the abdomen had greatly ſubſided. She did not long enjoy this flattering reſpite; in the ſpace of a week all the unfavourable ſigns returned, and within the enſuing ſix weeks ſhe was relieved by death from all her miſery.

I obtained leave to have the body opened.—On diſſection the appearances were as follow: The round and broad ligaments, and all the appendages of the womb, together with almoſt the whole of its ſubſtance, were conſumed; and there remained only a thin, moiſt, calcareous or earthy ſhell, which nearly enveloped a perfect fœtus. Theſe remains of the uterus reſted upon the ſacrum, having ſunk down to the bottom of the pelvis. The head of the child occupied the fundus uteri, the ſubſtance of which had been ſo entirely waſted away, that the hairy fcalp was left uncovered, and was feparating from the fubjacent cranium. The whole was fo putrid as to emit a moft intolerable ftench, and could not be preferved.

The vifcera of the abdomen were all of them found and perfect; and no ulceration could be perceived in any part of the alimentary canal, though carefully fearched after. On cutting into the great inteftines, they were found to be replete with the fame kind of fubftance, in a fluid ftate, as compofed the earthy tegument of the child in the pelvis.

We were not allowed to examine the contents of the head or thorax: but it is prefumed that both were found, as the patient's intellects were preferved to the laft, and there were no fymptoms of difeafed lungs * during her life. She was fix and thirty years of age; of a fair complexion; had been a ftout, well-formed woman; and had borne a child about feven years previous to her death.

* The dyfpnœa, mentioned amongft the other fymptoms, feems to have been owing to the preffure of the tumefied ftomach and abdomen againft the diaphragm, preventing its defcent and the free expanfion of the lungs, and not to any morbid affection of the latter.

I fear the melancholy cafe juft recited will not admit of much ufeful application, which is the grand aim of all refearch. One remark or two, however, I beg leave to fubjoin to this very fingular medical hiftory. A very obvious diftinction may be traced upon the face of it, between the wonted preparations which nature made to bring forth the infant, and the fymptoms which were produced by the accident. Let us make the comparifon. — The pain, in the different circumftances, was different in its degree and continuance. A difcharge, fuch as occurs previous to labour in all natural cafes, appeared alfo in this; but one of a very different kind iffued conftantly from the vagina, efpecially during the laft weeks of the patient's life. The inference to be drawn from this comparifon is this — that the efforts of nature are wonderful indeed, and deferve the higheft confidence of practitioners. In the prefent inftance, the uterus itfelf being difeafed, was unable to perform the functions of delivery, and, after fome faint attempts, its operations in the ufual way ceafed, whilft others were undertaken of a different kind indeed, but no lefs owing to the exertions of the *vis medicatrix*

*naturæ*. This will appear, perhaps, more clearly by attempting the probable pathology of the caſe. — It is not unlikely that a laceration of ſome of its ligaments, or of the womb itſelf, was the immediate effect of the poor woman's raiſing the pail of water to her head. Inflammation and ſuppuration enſued; the matter partly paſſed off *per vaginam*, and a part of it was alſo abſorbed and conveyed into the bowels, from whence it was diſcharged by ſtool. The thinner parts being firſt taken up by the abſorbents, the more ſolid earthy matter, which forms the baſis of the ſolida viva, remained to the laſt, forming the earthy tegument or ſhell above mentioned. Nature was buſied in removing this alſo, when, being exhauſted by too-long-continued exertions, ſhe was obliged to give up the ſtruggle.

*New Street, Spring Gardens,*
Jan. 24th, 1787.

VII. *Far-*

